# Prevalence and associated factors of malaria among pregnant women in Sherkole district, Benishangul Gumuz regional state, West Ethiopia

**DOI:** 10.1186/s12879-020-05289-9

**Published:** 2020-08-05

**Authors:** Girma Bekele Gontie, Haileab Fekadu Wolde, Adhanom Gebreegziabher Baraki

**Affiliations:** 1 Benishangul Gumuz Regional State Health Bureau, Asosa, Ethiopia; 2 Department of Epidemiology and Biostatistics, Institute of Public Health, College of Medicine and Health Sciences, University of Gondar, Gondar, Ethiopia

**Keywords:** Malaria, Pregnant women, Benishangul Gumuz, Ethiopia

## Abstract

**Background:**

Malaria during pregnancy leads to serious adverse effects on mothers and the fetus. Approximately 25 million pregnant women in sub-Saharan Africa live at risk of malaria. This study would help to achieve Sustainable Development Goals (SDGs) by improving programs that deal with the prevention of malaria. Therefore, this study aimed to assess the prevalence and associated factors of malaria among pregnant women.

**Methods:**

A community-based cross-sectional study was conducted from July to August 2018 in Sherkole district, West Ethiopia. A multi-stage sampling technique was used to select 504 pregnant women. The interviewer-administered semi-structured questionnaire was used for data collection. Malaria was also diagnosed using a rapid diagnostic test. The data was entered using EPI info version 7.2.2.2 and transferred to SPSS version 20 for analysis. Descriptive statistics were done using frequency and percentages. Both bivariable and multivariable logistic regression models were employed. Variables having *p*-value < 0.2 were included in the final multivariable model. Variables having *p*-values < 0.05 from the multivariable model were considered to be significantly associated with the dependent variable. The adjusted odds ratio with its 95% confidence interval (CI) was used as a measure of association.

**Results:**

Of the total 498 pregnant women who participated in this study, 51(10.2, 95% CI: 7.72–13.24) were found to have malaria. Of these, 46 (90.2%) and 5 (9.8%) were caused by *Plasmodium falciparum* and *Plasmodium vivax,* respectively. Decreasing Age (Adjusted Odds Ratio (AOR) 0.78; 95% CI 0.67–0.911), not using insecticide-treated bed net (ITN) (AOR 12.5; 95% CI 4.86–32.21), lack of consultation and health education about malaria prevention (AOR 7.18; 95% CI 2.74–18.81), being on second-trimester pregnancy (AOR 7.58; 95% CI 2.84–20.2), gravidae II (AOR 5.99; 95% CI 1.68–21.44) were found to be significantly associated with malaria during pregnancy.

**Conclusion:**

Malaria is still a public health problem among pregnant women in the Sherkole district. Age, ITN use, gravidity, gestational age, and health education had a significant association with malaria. Screening pregnant women for asymptomatic malaria infection and educating and consulting on the appropriate malaria preventive methods shall be provided.

## Background

Malaria is caused by parasites of the *genus Plasmodium* and transmitted by female Anopheles mosquitoes. There are five different human malaria species such as *P. falciparum*, *P. vivax*, *P. malariae, P. knowlesi* and *P. ovale*. In 2016, an estimated 216 million cases of malaria and 445,000 deaths occurred worldwide [1]. Most, (90%), malaria cases and 91% of all malaria death in 2015 and 2016 were reported from the WHO African Region. Of the 91 countries reporting indigenous malaria cases worldwide, around 80% of the total cases were from sub-Saharan African countries [1, 2].

Malaria during pregnancy is a serious public health problem in sub-Saharan Africa. It is estimated that each year approximately 25 million pregnant women in sub-Saharan Africa live at risk of *P. falciparum* malaria infection [3]. Two institution-based studies done among pregnant women attending antenatal care (ANC) in Nigeria showed the prevalence of malaria to be 41.6% [4] and 7.7% [5]. Another institution based study in Eastern Sudan showed 13.7% of pregnant women were infected with *P. falciparum* [6]. Studies conducted in Burkina Faso [7], and Malawi [8] also showed the prevalence to be 18.1%, and 19.% respectively. Besides, two institution and one community-based studies conducted in different parts of Ethiopia also showed the prevalence of malaria among pregnant women to be between 2.83 and 16.3% [9–11].

Malaria infection during pregnancy causes an enormous risk to the mother, fetus, and neonates [12]. Indeed although malaria during pregnancy might be asymptomatic due to a high level of acquired immunity in mothers residing in high transmission areas, it is still associated with an increased risk of maternal anemia, spontaneous abortion, stillbirth, prematurity, and low birth weight [3, 13, 14]. Moreover, severe maternal anemia increases the mother’s risk of death. Malaria-related anemia is estimated to cause as many as 10,000 maternal deaths each year in Africa [15].

Different risk factors for malaria among pregnant women were identified by previous studies. These include educational status [7, 16], age [5, 17], ANC visit, gestational age [18], parity [7, 18], gravidity, and ITN utilization [11].

In Benishangul Gumuz regional state, almost all districts (98%) of the landmass are malarious areas and 97% of the population are at risk for malaria infection. Despite the high risk of malaria transmission in the area, there is limited evidence about the burden and risk factors of malaria among pregnant women which can be used for reducing maternal and child mortality due to the disease. Therefore, this study aimed to assess the prevalence of malaria and its associated factors among pregnant women in Sherkole District, Benishangul Gumuz regional state, West Ethiopia.

## Methods

### Study area and period

**Fig. 1:**
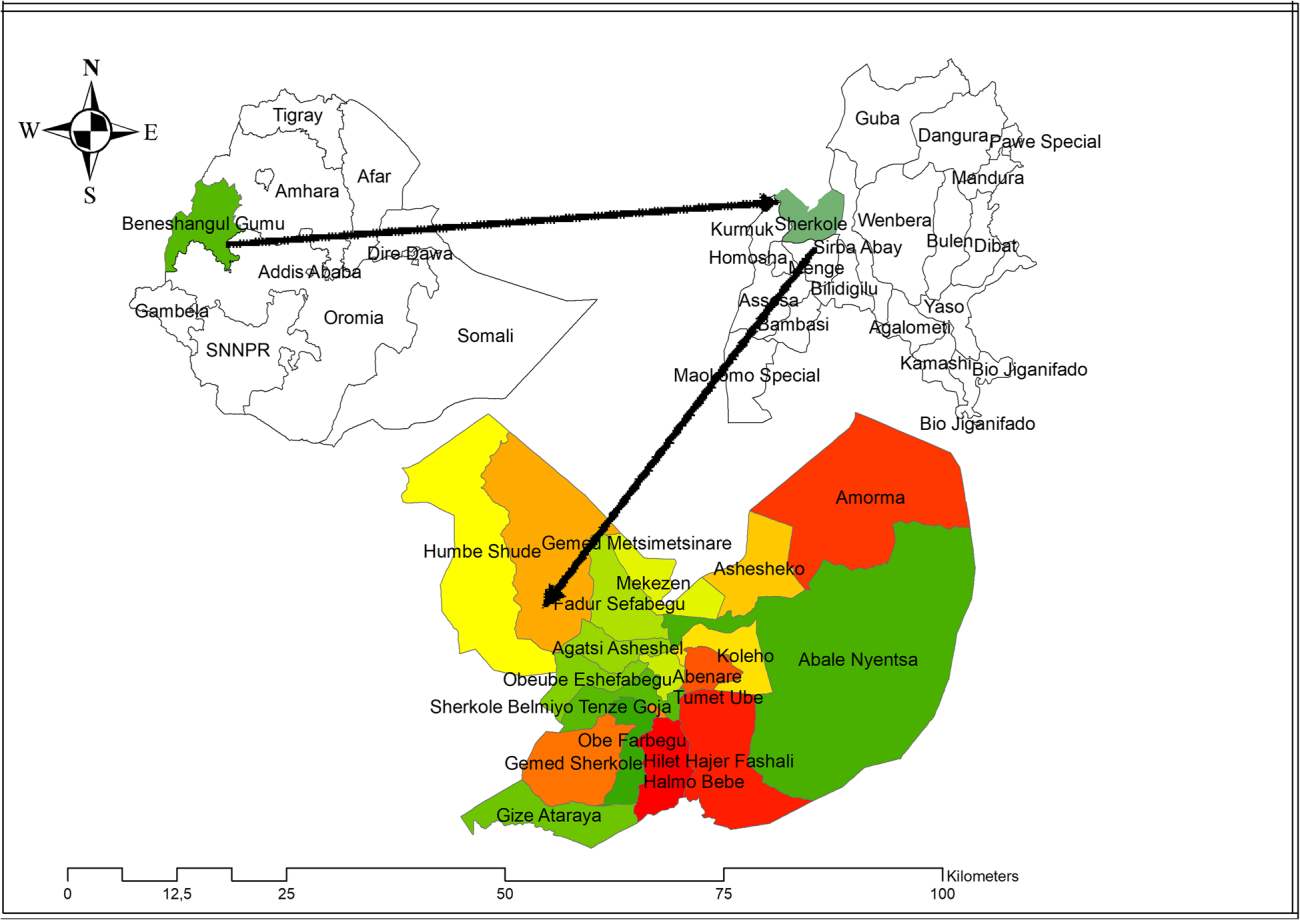
Location of the study area

The study was conducted in Sherkole district, Benishangul Gumuz Regional state (BGRS) from July 20 to August 30, 2018. Sherkole district is one of the 21 BGRS administration districts which is found 756 km to the West of Addis Ababa, the capital city of the country, and 96 km far away from region city, Assosa. The district is found at a latitude of 13.169308 and longitude of 39.987117 and the altitude of the district is 680–800 m above sea level. The climatic condition of the district is hot and the annual temperature is estimated to be between 25 °C and 41 °C. The Annual range of rainfall in the district is 900–1200 mm. In this district, all kebeles are malarious with 39,373 populations at risk of the disease. In 2016/2017, the annual malaria incidence rate in the district was 263 cases per 1000 population. There were 1243 pregnant women, 1196 under 1 year, and 6370 under 5 years old of children in the district (Fig. 1).

### Study design and population

A community-based cross-sectional study was conducted. The source population for this study was all pregnant women at any gestational age living in the district. The study population was those pregnant women in the selected kebeles and who were available during the data collection period. Pregnant women with mental illness and severely debilitating diseases were excluded from the study.

### Sample size determination and sampling procedure

The sample size was determined using a single proportion formula using a 50% prevalence of malaria among pregnant women, 95% confidence level, 5% margin of error, and design effect of 2. To compensate for the non-response rate, 10% of the determined sample size was added. Finally, finite population correction was done to adjust the final sample size which gives a total sample size of 504. A multi-stage sampling technique was used to select the determined sample size. At the first stage, from a total of 20 kebeles in the district, 8 kebeles with a a total of 1243 pregnant women were selected by using a simple random sampling technique. In the second stage, the sample size was distributed proportionally for the 8 kebeles based on the number of pregnant women in the kebeles with a range of 41 to 79 housholds for each kebele and then households were selected using a simple random sampling technique. Finally, pregnant women in the household were taken and in the presence of more than one eligible woman in a single household, a lottery method was used to select one.

### Variable measurement and data collection procedure

The outcome variable for this study was malaria infection which was assessed using RDT and pregnant mother with any type of *Plasmodium* species from the test were considered as having malaria infection. The independent variables include socio-demographic factors (age, marital status, educational status, and occupational status); obstetric factors (gravidity, parity, trimester of pregnancy, history of abortion); malaria prevention measures (ITN ownership, indoor residual spraying (IRS) use, personal protective measures, and ITN utilization); health service use (accessibility of ANC, gestational age at the first visit, number of ANC visit, place of delivery for the previous child, previous history of malaria infection during pregnancy, and health education about malaria prevention methods during ANC follow up).

The interviewer-administered Semi-structured questionnaire was used to collect the required information. For those pregnant women who were on ANC followup, the data collector reviewed their antenatal followup cards to cross-check the information given by them. Card information checked includes; gravidity, parity, and gestational age at first ANC visit. Following the interviews, blood was obtained from the third finger of women’s left hand. First, the tip of the finger was wiped with a piece of cotton wool lightly soaked in alcohol. Then piercing with sterile lancet was done and the blood allowed to flow freely without squeezing the finger. Then, 5 μl (μl) blood was collected and a single small drop was added on the CareStart RDT to examine the presence or absence of malaria and to differentiate its species. The RDT read and determine the species qualitatively after 15–20 min of putting the blood to the kit. Ten percent of the randomly selected negative slides were rechecked and reread. Eight trained diploma nurses and midwives collected the data and they were supervised by two health professionals with a qualification of BSc degree. The questioner was pretested and one-day training was given for supervisors and data collectors on the basic technique of the data collection.

### Data processing and analysis

The data were entered using EPI-Info 7.2.2 and then transferred to SPSS version 20 statistical package for further analysis. Data cleaning and management were done. Descriptive statistics (frequencies, mean, SD, and percentage) were done to explain the study population in relation to relevant variables. The Chi-square assumption was checked for all categorical independent variables and multicollinearity was also checked using the Variance inflation factor (VIF). Both bi-variable and multi-variable logistic regressions were used to assess the association between outcome and explanatory variables. Factors with *p*-value ≤0.2 from the bi-variable model were included in the final model. Variables having a *p*-value < 0.05 from the multivariable model were considered as having a statistically significant association with the outcome. Adjusted Odds ratio with 95% CI was used as a measure of association. The model goodness of fit was assessed using the Hosmer lemisho test.

## Result

### Socio-demographic characteristics, obstetric characteristics, and malaria prevention methods adopted by pregnant women

A total of 498 pregnant women participated in this study with a response rate of 98.8%. The majority, 208(41.8%), of the pregnant women were in the age group of 25–29 years. Concerning the educational status, more than three fourth, 384(77.1%), of the mothers had no formal education. Almost all, 482 (96.8%), study participants were farmers and traditional gold miners. About 478 (96%) of respondents owned at least one mosquito bed net, and 405 (81.3%) of them sleep under mosquito nets in the previous night. Almost all, 485 (97.8%), of the households had Indoor Residual Spray (IRS) in the last 12 months. All women 431 (86.5%) who had ANC follow-up were given health education about the prevention methods of malaria infection during their ANC follow-up. The majority, 323 (64.9%), of the study participants were multi-gravida, and more than half, 292 (58.6%), of the study participants were in their third trimester of pregnancy (Table 1).

**Table 1:** Socio demographic characteristics and malaria prevention methods in Sherkole district, West Ethiopia, 2018

Description	Frequency	Percent (%)
**Age group (Years)**		
15–19	14	2.8
20–24	87	17.5
25–29	208	41.8
30–34	122	24.5
> =35	67	13.5
**Marital status**		
Married	492	98.8
Others ^a^	6	1..2
**Occupation**		
Farmer and traditional gold mining	482	96.8
Civil servants	12	2.4
Merchant	4	0.8
**Education**		
No formal education	384	77.1
Primary	109	21.9
Others^b^	5	1
**ITN ownership**		
Yes	478	96
No	20	4
**ITN Utilization**		
Yes	354	71.3
No	93	18.7
**Use IRS in the last 12 months**		
Yes	485	97.8
No	13	2.2
**Consult and given health education about malaria prevention and control method**		
Yes	431	86.5
No	67	13.5
**Gravidity**		
Primigravidae	51	10.2
Secundigravidae	124	24.9
Multigravidae	323	64.9
**Trimester**		
1st	15	3
2nd	191	38.4
3rd	292	58.6
**previous place of delivery**		
At Health facility	296	66.2
At home	151	33.8
**ANC visit**		
No ANC follow up	67	13.5
1 time	163	32.8
2 time	158	31.8
3 time	94	18.9
4+ time	15	3

*ANC* Antenatal care, *IRS* Indoor Residual Spray

^a^ show divorced, single and widowed, ^b^show higher education and Technical and vocational collage

### Prevalence of malaria infection among pregnant women

In our study, the prevalence of malaria was found to be 10.2% (95% CI: 7.72–13.24). Of these, 46(90.2%) were *P. falciparum* cases and 5 (9.8%) were *P. vivax* cases. From the total confirmed cases, the majority, 35 (68.8%) were asymptomatic.

### Factors affecting malaria infection among pregnant women

From the bi-variable logistic regression, malaria was significantly associated with all of the variables at a significance level of 0.2. However, from the multivariable logistic regression model only age, ITN utilization, consultation about malaria prevention methods during ANC, trimester of pregnancy, and gravidity were significantly associated with malaria infection during pregnancy. For 1 year increase in the age of the pregnant women, the odds of malaria infection was decreased by 22%(AOR = 0.78, 95% CI: 0.67, 0.91). The odds of malaria infection was 14.98 times higher among pregnant women who did not utilize ITN compared to their counterparts (AOR = 14.98, 95% CI: 5.24, 42.27). Pregnant women who had no education about malaria prevention methods during their ANC follow up had 7.15 times increased odds of malaria infection compared to their counterparts (AOR = 7.15, 95% CI: 2.44, 20.96). Women who were in their first trimester of pregnancy had 23.33 times increased odds of having malaria infection compared to mothers on their third trimester (AOR = 23.33, 95% CI: 1.90, 28.20). Women who are in their second trimester of pregnancy also had 7.78 times increased odds of having malaria infection compared to mothers on their third trimester (AOR = 7.78, 95% CI:2.77, 21.87). The odds of malaria infection was 5.87 times higher among women who had their second pregnancy compared to multi gravid women (AOR = 5.87, 95% CI: 1.61, 21.37) (Table 2).

**Table 2:** Multivariable analyses of factors associated with malaria infection among pregnant women in Sherkole district, Benishangul Gumuz regional state, West Ethiopia, 2018

Explanatory variable	Malaria status		Crud OR(95%CI)	Adjusted OR (95%CI)	P-value
	Positive(%)	Negative(%)			
***Age***			0.74 (0.681, 0.812)	0.78 (0.67, 0.91)	0.001
***ITN ownership***					
Yes	42 (8.8)	436 (91.2)	1	1	
No	9 (45.0)	11 (55.0)	8.49 (3.33, 21.66)	0.49 (0.09, 2.60)	0.401
***ITN utilization***					
Yes	17 (4.2)	388 (95.8)	1	1	
No	34 (36.6)	59 (63.4)	13.15 (6.91, 25.03)	14.89 (5.24, 42.27)	0.000
***ANC follow up***					
Yes	28 (6.5)	404 (93.5)	1	1	
No	23 (34.9)	43 (65.1)	7.72 (4.09, 14.56)	0.91 (0.27, 3.12)	0.887
***consultation and education about the prevention methods of malaria during ANC***					
Yes	24 (5.57)	407 (94.4)	1	1	
No	27 (40.3)	40 (59.7)	11.45 (6.04, 21.68)	7.15 (2.44, 20.96)	0.000
***Trimester***					
1st	3 (20.0)	12 (80.0)	8.88 (2.08, 37.73)	23.33 (1.90, 28.20)	0.014
2nd	40 (20.9)	151 (79.1)	9.4 (4.29, 20.6)	7.78 (2.77, 21.87)	0.000
3rd	8 (2.7)	284 (97.3)	1	1	
***Gravidity***					
Premigravidea	12 (23.5)	39 (76.5)	12.11 (4.67, 31.47)	0.95 (0.14, 6.56)	0.963
Secundgravidea	31 (25.0)	93 (75.0)	13.13 (5.83, 29.53)	5.87 (1.61, 21.37)	0.007
Multigravidea	8 (2.5)	315 (97.5)	1	1	

## Discussion

This study assessed the prevalence of malaria infection and associated factors among pregnant women in Sherkole district, Benishangul Gumuz regional state, West Ethiopia. Different studies reported different factors that affect the rate of malaria infection among pregnant women. Our study also assessed socio-demographic, obstetric, and ITN ownership and utilization factors. As a result, Age the woman, ITN utilization, health education about prevention methods during pregnancy, gestational age, and gravidity were found to be significantly associated with malaria infection.

In this study, the prevalence of malaria was found to be 10.2%. This result was higher than studies conducted in Felege Hiwot referral hospital and Addis Zemen health center, Ethiopia (2.83%) [9], rural district surrounding Arbaminch town, Ethiopia (9.1%) [11], coastal Ghana (5%) [19], South-West Nigeria (7.7%) [5], Southern Laos (8.3%) [20] and India (5.4%) [21]. This difference might be attributed to the difference in geographical location among the study areas. For instance, our study was conducted in a malaria-endemic area with a high rate of transmission. Therefore, individuals living in malaria-endemic areas have a greater chance of developing asymptomatic malaria, while those living in low transmission areas have a low chance of being infected, which can lead to a low prevalence of the diseases in such areas. Another reason for the difference could be the inclusion criteria used by the studies because our study included both symptomatic and asymptomatic pregnant women which might increase the prevalence but most of the other studies included only asymptomatic pregnant women. On the other hand, the prevalence in our study was found to be lower than studies conducted in Pawe hospital, Ethiopia (16.3%) [10], Sudan (13.7%) [6], Nigeria (41.6%) [4], Malawi (19.6%) [8] Burkinafaso (18.1%) [7] and a systematic review and meta-analysis in Ethiopia 12.7% [22]. It is also found to be much lower than the findings from two studies conducted in Nigeria which showed the prevalence to be 58% [23] and 59.9% [24]. This difference may be due to better implementation of improved malaria interventions including increased coverage in the distribution of Long Lasting Insecticide Treated Nets (LLINs), and indoor residual spraying in our study area which showed 96 and 81.3% of respondents own and utilize ITN, respectively. Almost all (98%) of participants in our study area also lived in residual sprayed households. Therefore, these interventions might reduce the malaria burden in the study area. Another possible reason for the low prevalence in our study could be, the study was done during the low malaria transmission season (July – August). However, the major transmission for malaria occurs between September and December.

In this study, 90.2% of the cases were caused by *P.falciparum* species. This result was in line with the study conducted in tropical Africa which showed 80–95% of malaria infections are caused by *P. falciparum* [19]. However, our result was higher than the national prevalence reports of the species which was 60–70% [25]. This high proportion of this malaria species in our study is a clear implication that there is a need for aggressive prevention and control of the diseases, especially among pregnant women. Because *P. falciparum* causes the most severe form of the disease and it can cause devastating complications not only for the mother but also for the fetus. This result also implies that there is a need for early screening of pregnant women for early detection and treatment of the cases to prevent possible complications. On the other hand, the proportion of malaria cases caused by *P.falciparum* in our study was lower than the WHO malaria 2017 report which revealed over 99% of malaria cases were due to *P.falciparum* [1]. The possible reason for these variations might be due to marked seasonal, inter-annual, and spatial variability. It may also be due to large differences in climate (temperature, rainfall, and relative humidity), human settlement, and population movement patterns.

In this study mothers with an increased age were found to have lower odds of developing malaria infection. This is in line with studies conducted in different tropical African countries [5, 17] which reported pregnant women of young age are at the greatest risk of malaria infection, as well as having the highest parasite densities. This may be attributed to mothers with increased age have better exposure to health services and gain a good awareness about the disease and the ways of prevention. Also, due to previous frequent malaria exposures, older aged mothers might develop immunity to malaria. However, according to the studies conducted in rural surroundings of Arbaminch Town, Ethiopia [11], and Sudan [6], age had no significant association with malaria infection.

According to our study, pregnant women who were in the second trimester of pregnancy were at increased odds of developing malaria infection compared to mothers in the third trimester. Besides, and women who were gravidae II have increased odds of malaria infection compared to the multi gravid. Similar results were found from studies done in sub-Saharan Africa countries [7, 11, 18], which showed a higher risk of malaria infection among primigravidae and gravida two than multigravidae. Low risk of malaria among multigravidae mothers may be associated with the development of pre-immunity to malaria with increased gravidity and previous exposures. It might be also linked to infection-specific immunological factors. Some *Plasmodium*-infected erythrocytes sequester/arrest in the maternal placenta by producing surface antigens mainly variant surface antigens that adhere to chondroitin sulphate-A (CSA) receptors expressed by syncytiotrophoblasts in the placenta. These antibodies are associated with protection against placental infection. Therefore, primigravidae and secundigravidae mothers lack these anti-adhesion antibodies against CSA binding parasites, which develop only after successive pregnancies and this makes them more susceptible to infection [26].

In our study, getting a consultation and health education about malaria preventive methods during ANC follow up significantly decreased the odds of developing malaria infection during pregnancy. A similar association was found in studies conducted different parts of Ethiopia [27, 28]. Health education and consultation specifically on prevention and control program of malaria during pregnancy ensures the use of antimalarials and other intervention measures effectively.

In this study, not using ITN increases the odds of developing malaria infection during pregnancy. Indeed, WHO, MoH, and presidents malaria initiatives (PMI) have advocated for a three-pronged approach to tackle malaria and one of the strategies is the use of ITN [1, 25, 29]. This study’s finding was also in agreement with the study conducted in Malawi [8], Nigeria [30], and Arbaminch, Ethiopia [11], which showed that the use of bed nets has a significant impact on decreasing malaria infection. The possible explanation for this association could be ITNs effectively reduce human-mosquito contact which can prevent diseases.

Since our study used a cross-sectional study design, it does not show a direct temporal relationship. Though using PCR and blood film microscopy may have higher sensitivity, we could not do these tests because the study is done in rural areas and there is no electricity in the area. Therefore, the result of this study could be affected by the inherent performance of the RDT utilized.

## Conclusion

The prevalence of malaria infection among pregnant women was relatively low in Sherkole district and *P. falciparum* is the most predominant *Plasmodium* species in the area. Age of respondents, ITN use, gravidity, gestational age, and health education about malaria prevention methods during ANC had a significant association with malaria infection. Health professionals should give health education about malaria prevention methods during ANC and they should also give special attention to those pregnant women with the identified risk factors. Besides, further research is recommended by using more sensitive diagnostic methods like PCR and blood film microscopy for the diagnosis of malaria.

## Data Availability

The data upon which the result based could be accessed a reasonable request.

## Abbreviations

AOR: Adjusted Odds Ratio
ANC: Antenatal Care
BGRS: Benishangul Gumuz Regional state
(CSA): Chondroitin Sulphate-A
CI: Confidence Interval
ITN: Insecticide Treated Net
LLINs: Long Lasting Insecticide Treated Nets
MoH: Ministry of Health
OR: Odds Ratio
PMI: Presidents Malaria Initiatives
RDT: Rapid Diagnostic Test
WHO: World Health Organization
